# Cartilage-Specific Overexpression of ERRγ Results in Chondrodysplasia and Reduced Chondrocyte Proliferation

**DOI:** 10.1371/journal.pone.0081511

**Published:** 2013-12-09

**Authors:** Marco Cardelli, Ralph A. Zirngibl, Jonathan F. Boetto, Kristen P. McKenzie, Tammy-Claire Troy, Kursad Turksen, Jane E. Aubin

**Affiliations:** 1 Department of Medical Biophysics, University of Toronto, Toronto, Ontario, Canada; 2 Department of Molecular Genetics, University of Toronto, Toronto, Ontario, Canada; 3 Regenerative Medicine Program, Sprott Centre for Stem Cell Research, Ottawa Hospital Research Institute, Ottawa, Canada; University of Texas Southwestern Medical Center, United States of America

## Abstract

While the role of estrogen receptor-related receptor alpha (ERRα) in chondrogenesis has been investigated, the involvement of ERR gamma (ERRγ) has not been determined. To assess the effect of increased ERRγ activity on cartilage development *in vivo*, we generated two transgenic (Tg) lines overexpressing ERRγ2 via a chondrocyte-specific promoter; the two lines exhibited ∼3 and ∼5 fold increased ERRγ2 protein expression respectively in E14.5 Tg versus wild type (WT) limbs. On postnatal day seven (P7), we observed a 4–10% reduction in the size of the craniofacial, axial and appendicular skeletons in Tg versus WT mice. The reduction in bone length was already present at birth and did not appear to involve bones that are derived via intramembranous bone formation as the bones of the calvaria, clavicle, and the mandible developed normally. Histological analysis of P7 growth plates revealed a reduction in the length of the Tg versus WT growth plate, the majority of which was attributable to a reduced proliferative zone. The reduced proliferative zone paralleled a decrease in the number of Ki67-positive proliferating cells, with no significant change in apoptosis, and was accompanied by large cell-free swaths of cartilage matrix, which extended through multiple zones of the growth plate. Using a bioinformatics approach, we identified known chondrogenesis-associated genes with at least one predicted ERR binding site in their proximal promoters, as well as cell cycle regulators known to be regulated by ERRγ. Of the genes identified, *Col2al*, *Agg, Pth1r*, and *Cdkn1b (p27)* were significantly upregulated, suggesting that ERRγ2 negatively regulates chondrocyte proliferation and positively regulates matrix synthesis to coordinate growth plate height and organization.

## Introduction

The bones of the axial and appendicular skeleton arise from condensations of chondrogenic cells that lay down a cartilaginous scaffold, which is then remodeled to give rise to the ossified bone. The longitudinal growth of endochondral bones is driven by continued chondrogenesis in the growth plate, which can be divided along the longitudinal axis of the bone into distinct zones comprising resting, proliferating, and post-mitotic chondrocytes, respectively, from the articular surface. The process of chondrogenesis is a highly orchestrated proliferation-differentiation sequence that is regulated by a number of signaling pathways and feedback loops. For example, the homeobox transcription factor, SRY-related high-mobility-group box 9 (SOX9), is the primary determinant of chondrogenesis and is required for the initial commitment of mesenchymal stem cells to the chondrogenic lineage [1]. The hedgehog protein family member, Indian hedgehog (IHH), is also crucial to chondrocyte proliferation and differentiation. It is expressed by prehypertrophic cells and binds to its receptor Patched-1 (PTC-1), which, in turn, activates signaling pathways to promote chondrocyte proliferation [2]. IHH also forms a negative regulatory feedback loop with parathyroid hormone-related protein (PTHRP) to delay chondrocyte hypertrophy and increase the pool of proliferating chondrocytes [3]. On the other hand, the transcription factor RUNX2 promotes the differentiation of chondrocytes from proliferation to hypertrophy [4].

Certain transcription factors belonging to the nuclear hormone receptor family are also involved in chondrocyte differentiation. These include the two estrogen-binding receptors, estrogen receptor alpha and beta (ERα (NR3A1) and ERβ (NR3A2) respectively), and recent reports highlight a role for ERα in the fusion or slowing down of growth plate chondrogenesis at puberty in humans and mice. For example, in a cartilage-specific ERα-deleted mouse, appendicular bones developed normally, but exposure to high levels of estrogen failed to reduce bone length as it did in wild type (WT) mice, indicating that ERα was required for the natural deceleration of bone growth that occurs in mice upon sexual maturity [5]. Conversely, a mouse line that expressed a constitutively active form of ERα in cartilage exhibited fewer proliferating cells in the growth plate and reduced bone length [6].

Three orphan nuclear receptor genes related to the ERs comprise the estrogen receptor-related receptor (ERR) family: alpha, beta and gamma (NR3B1, NR3B2, and NR3B3, respectively) [7]. These genes share a high degree of similarity with the ERs, including 67% identity in the DNA-binding domain (DBD), but are unable to bind estrogen [8]. With their similarity in their DBD, it is not surprising that there is considerable cross-talk at the level of gene regulation between the ERs and the ERRs. However, X-ray crystallography studies have clearly shown that, unlike the ERs, the ERRs assume an active state without a ligand bound to the ligand binding domain (LBD) [9], [10]. Consistent with the hypothesis that the ERRs are constitutive transcriptional activators, *in vitro* transcription assays demonstrated that ERRα and ERRγ induce expression of target genes without addition of potential ligand to the media [11], [12]. *ERRα−/−* mice display a significant decrease in body mass and *ERRγ−/−* mice are perinatal lethal due to cardiac failure [13], [14], phenotypes connected to the roles that both of these isoforms play in energy metabolism.

The role of ERRs in bone and cartilage are also beginning to be investigated, with most data published on ERRα [15]. ERRα is expressed in proliferating chondrocytes *in vivo* and throughout chondrocyte differentiation *in vitro* [16]. In addition, it has been shown that ERRα is dysregulated in murine models of inflammatory arthritis [17], as well as in human osteoarthritis [18]. There is no data in the literature describing ERRγ in cartilage, and only very little described on its role in bone. Results of an epidemiological study of *ERRγ* polymorphisms in humans indicated a correlation between a subset of *ERRγ* variants and elevated bone mass [19]. *In vitro* overexpression of ERRγ causes a decrease in the expression of bone sialoprotein (*Bsp*) and osteocalcin (*Ocn*), markers of mature osteoblasts, in the MC3T3 pre-osteoblast cell line [20]. Taken together, these results suggest that ERRγ is a negative regulator of osteogenesis. To determine whether ERRγ also has a biologically relevant function in cartilage, we have generated transgenic (Tg) mice with a collagen α1 (II) (Col2) promoter driving expression of a full length ERRγ2 (long isoform) transcript (Col2::ERRγ2FL). We report here that overexpression of ERRγ2 in a cartilage-specific manner leads to abnormalities in the axial and appendicular skeletons.

## Results

### Overexpression of ERRγ2 Results in Dwarfism

**Figure 1:**
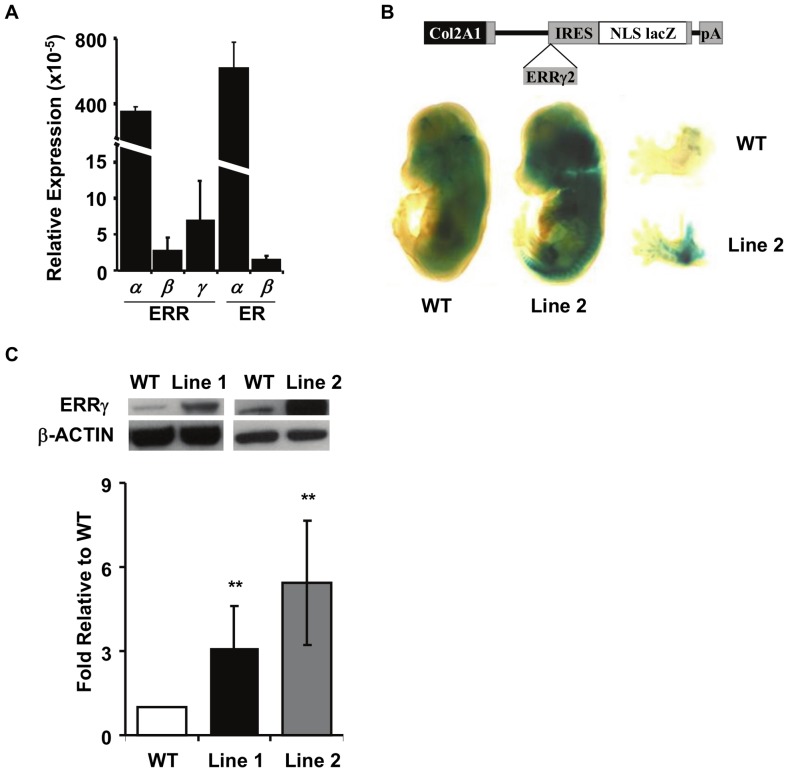
Col2::ERRγ2 transgenic mouse generation and protein expression analysis. (A) ERR and ER gene expression (relative to L32) in adult mouse cartilage. Graphs represent mean ± SD from a minimum of 3 independent samples. (B) Schematic of the transgene used to express the long protein isoform, ERRγ2, in cartilage. Whole mount lacZ staining of a WT and transgenic 14.5 dpc embryo with a close-up of the forelimbs showing robust staining in the developing cartilage. Some nonspecific staining along the neural tube is evident. (C) Western blots and resulting quantification showing the levels of ERRγ and β-ACTIN proteins in 14.5 dpc limbs of the two transgenic lines compared to WT. Graphs represent the mean ± SD from at least three independent Western blots. **p≤0.01.

To begin to investigate the putative role of ERRγ in chondrogenesis, we first asked whether ERRγ is expressed in cartilage. We found that *ERRγ* is expressed in mouse cartilage at levels similar to *ERRβ* and *ERβ*, but approximately 50 fold less than *ERRα,* and 85 fold less than *ERα* (Figure 1A). To evaluate the consequences of cartilage-specific overexpression of ERRγ, we generated two independent Tg mouse lines, Line 1 (#6486) and Line 2 (#4094), that express the longer protein isoform of ERRγ2 under the control of the collagen α1 (II) promoter (Col2::ERRγ2FL) (Figure 1B). LacZ staining of 14.5dpc embryos revealed distinct staining in the developing craniofacial skeleton and both the axial and appendicular skeletons of Tg mice but not wild type (WT) embryos (Figure 1B). Western blot analysis of 14.5 dpc limbs demonstrated increased expression of ERRγ2 protein relative to the β-ACTIN control and quantification demonstrated approximately 3-fold overexpression of ERRγ2 in Line 1, and 5-fold overexpression in Line 2 (Figure 1C). Mice were born in the expected Mendelian ratios and up to at least 8 months of age appeared healthy, suggesting that there is no overt detriment due to the integration or expression of the transgene.

**Figure 2:**
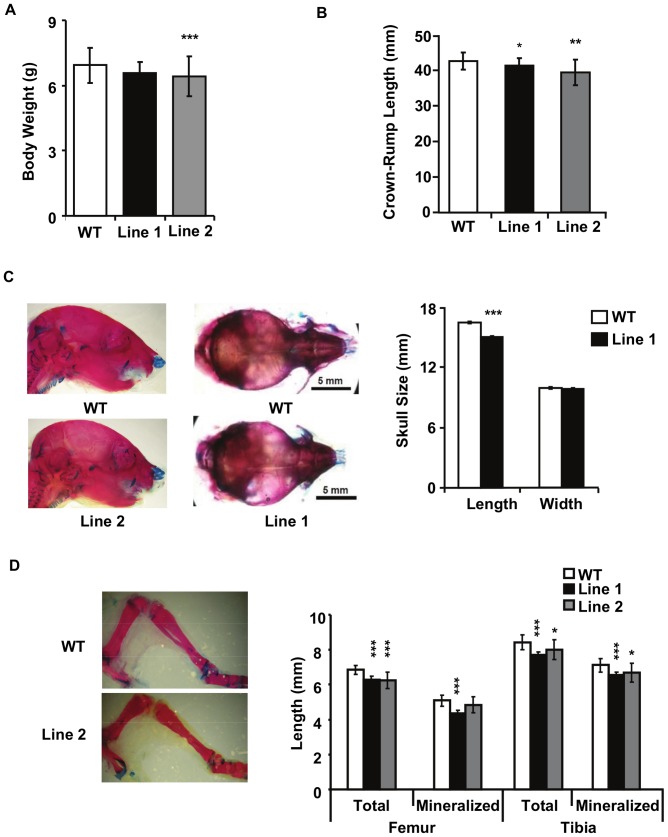
Skeletal analysis of P7 animals. (A) Body weight measured in P7 pups from WT (n = 19), Line 1 (n = 11) and Line 2 (n = 9) animals. (B) Crown-rump length measurement showed reduced axial skeleton. Measurements were taken from the snout to the base of the tail from WT (n = 24), Line1 (n = 16) and Line 2 (n = 13) pups. (C) Close-up photographs of the skulls from WT and Tg animals, and resulting quantification showing reduced length, but not width, in Tg compared to WT animals, WT (n = 6) and Line 1 (n = 11) pups. (D) Alcian blue/Alizarin red double stain of WT and Line 2 hindlimbs. Quantification shows reduced bone length and mineralized component of the axial skeleton in Tg compared to WT animals. Total femur and tibia length were measured as well as the length of the mineralized portion of the bone as demarcated by the Alizarin red staining for WT (n = 22), Line 1 (n = 14), Line 2 (n = 14). Graphs represent the mean ± SD *p≤0.05, **p≤0.01, ***p≤0.001.

Significant anomalies in all endochondral bones studied and measured were already apparent at birth in Tg versus WT mice (data not shown), but data are reported here only for postnatal day 7 (P7). Measurement of P7 pup weight showed no significant difference in Line 1, but a significant reduction in Line 2 compared to WT mice (Figure 2A). Quantification revealed a small but significant decrease in total body length (data not shown) as well as in the crown-rump length (Figure 2B) in pups of both lines. In P7 whole mount skeletal preparations, Tg mice also exhibited a shortened snout and domed head (Figure 2C) and a significant decrease in skull length, but not width compared to WT mice (Figure 2C). Analysis of specific long bones showed a reduction in the length of the femur and the tibia (Figure 2D) in both lines, as well as the humerus, ulna, radius and scapula (length but not width was affected) (data not shown). No significant differences were observed in Tg versus WT bones that form by intramembranous ossification, e.g., the mandible and the clavicle (data not shown). To rule out the possibility that the phenotypes we observed were due to the sex of the pups, we genotyped them for gender, and found that the phenotypes we describe occur in both male and female mice. Taken together, the data indicate that targeted ERRγ overexpression in cartilage results in mild dwarfism.

### Overexpression of ERRγ Impairs Chondrocyte Proliferation, Differentiation-maturation, Cartilage Matrix Production and Growth Plate Organization

**Figure 3:**
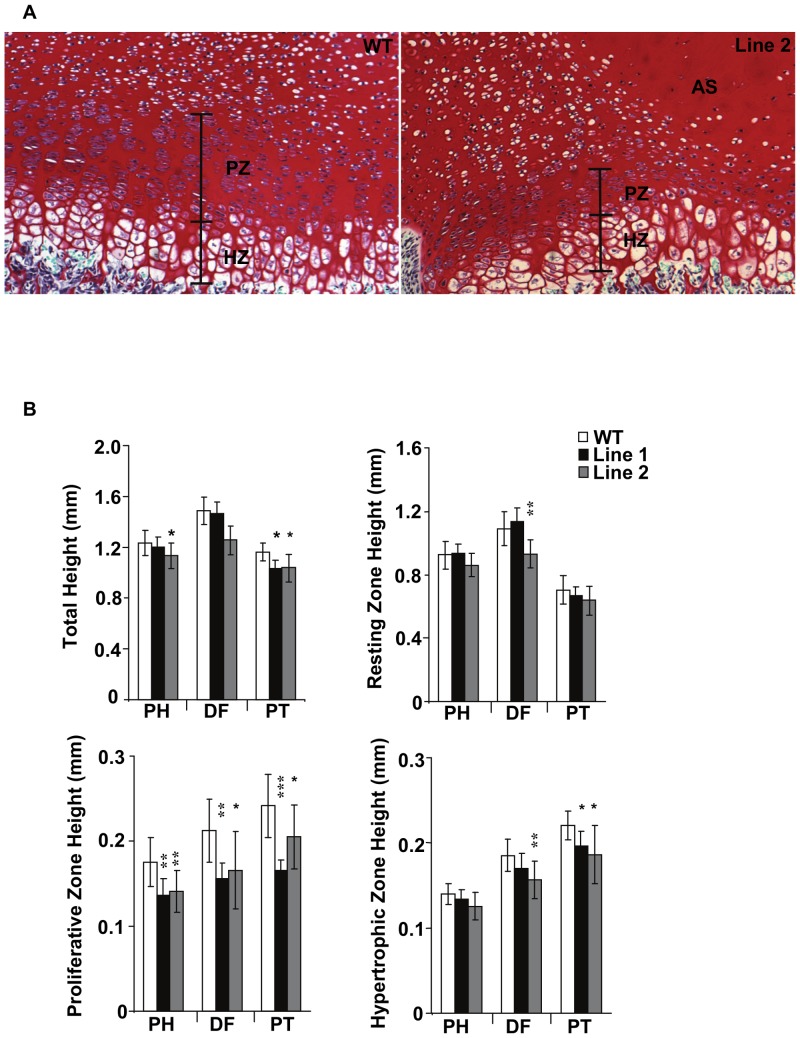
Growth plate analysis of P7 pups. (A) Growth plates from WT and Line 2 femurs showing the reduced proliferative zone and the acellular swath observed in the transgenic animals. (B) Analysis of total, resting, proliferating, and hypertrophic growth plate heights from proximal humerus (PH), distal femur (DF) or proximal tibia (PT). The largest decrease in growth plate height was observed in the proliferating zones of Tg animals compared to WT. *p≤0.05, **p≤0.01, ***p≤0.001 RZ, Resting Zone; PZ, Proliferative Zone; HZ, Hypertrophic Zone; AS, Acellular swath.

Quantification of P7 proximal humerus, distal femur, and proximal tibia revealed trends or significant decreases in total growth plate height as well as the proliferative and hypertrophic zones in Tg versus WT mice (Figure 3A and B). The most dramatic decrease was in the proliferative zone, which displayed a 22% height reduction in the transgenic versus WT mice, whereas hypertrophic zone changes were less pronounced and not detectable in all bones (Figure 3B). The quantification of zone heights in the Tg mice was complicated by 2 factors: a smaller and disorganized proliferative zone, and the presence of acellular swaths of cartilage matrix that often spanned the resting, proliferative and hypertrophic zones (Figure 3A). Although we observed 2 WT samples containing acellular swaths, such areas were much smaller and less pronounced than those in Tg samples.

**Figure 4:**
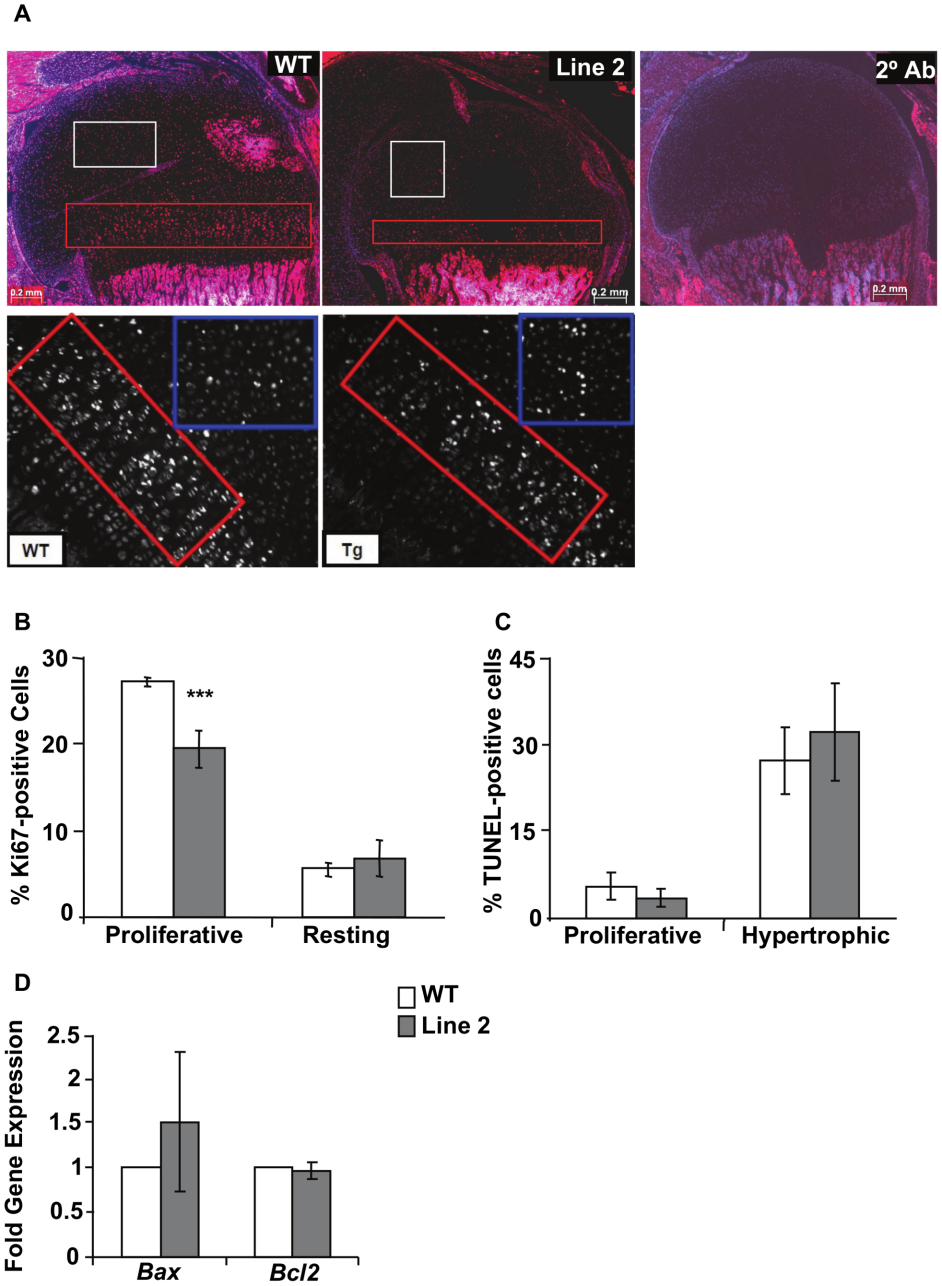
Decreased proliferation, but not apoptosis, is responsible for reduction of growth plate height. (A) Hoechst labeling and immunofluorescence of proximal humerus growth plate from WT and Line 2 samples, showing Ki67 positive cells in the proliferating zone (PZ) (red boxes), and resting zone (RZ) (white box). 2° Ab is a section stained with secondary antibody only and Hoechst. Lower panel shows the proliferative zone (red box) and part of the resting zone (blue box) under higher magnification, highlighting the difference in Ki67 positive cells within the proliferative zone of WT and Tg mice (B) Quantification of Ki67 positive cells from Line 2 proximal humerus growth plates demonstrated a clear reduction in the number of proliferating cells in the proliferating zone compared to the resting zone. WT (n = 6), Line 2 (n = 6); ***p≤0.001 (C) Quantification of TUNEL positive cells shows no difference in apoptotic cells in either the proliferating or hypertrophic zones. (D) Gene expression for apoptotic markers, *Bax* and *Bcl2,* from 14.5 dpc limbs confirms lack of increased apoptosis.

To determine the basis of the reduction in growth plate zone heights, we quantified the number of proliferating cells by immunostaining for the proliferation marker, Ki67 (Figure 4A). No significant difference was observed in resting zone chondrocytes, but a 30% decrease in Ki67-positive cells was seen in the Tg compared to WT proliferative zone (Figure 4B). Since acellular masses of matrix have been reported previously in other genetically-modified mice, including ones with unusually wide or generally disorganized growth plates in which hypoxic cell death occurs [1], we next performed a TUNEL assay. No significant difference in TUNEL staining was observed in either the hypertrophic or proliferative zones of Tg versus WT growth plates (quantified for Line 2; Figure 4C). RT-qPCR also revealed no differences in expression of the apoptosis-associated markers, *Bax* and *Bcl2*, in RNA isolated from the growth plate of WT versus Tg mice (Figure 4D). The data suggest that the reduced length of the proliferative zone is a consequence of decreased chondrocyte proliferation, whereas that of the hypertrophic zone may be due to a disruption in chondrocyte differentiation, a delay in chondrocyte maturation, or secondary effects to the reduction in proliferation.

**Figure 5:**
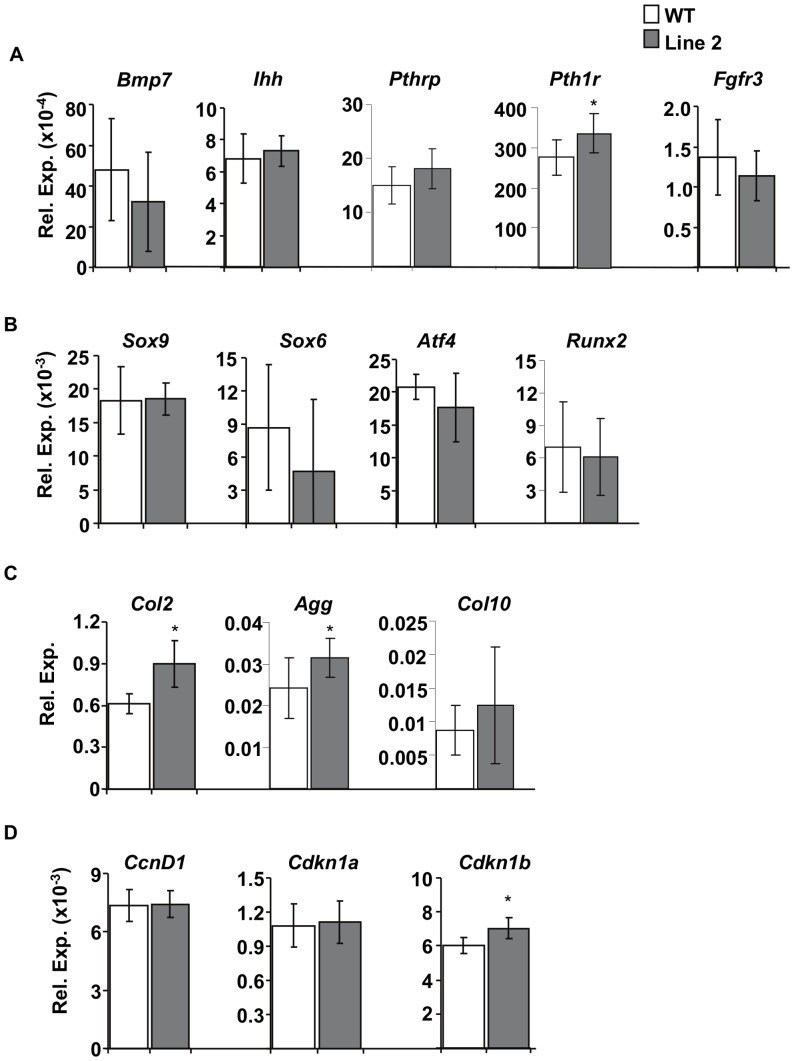
Expression profile of putative target genes. Genes are grouped as extracellular growth factors (A), transcription factors (B), ECM proteins (C), and cell cycle regulators (D). Of the genes tested, we observed increased *Pth1r*, *Col2a1, Agg,* and *Cdkn1b* expression in 14.5 dpc limbs of Tg compared to WT animals. Expression by RT-qPCR of putative target genes was normalized to the expression of L32. WT (n = 7), Line 2 (n = 9), *p≤0.05.

To investigate the molecular basis of changes observed in the growth plate as a consequence of overexpression of ERRγ2, we next used an *in silico* database search to screen for putative ERR binding sites in the region spanning 10 Kb upstream to 5 Kb downstream of the transcriptional start site in a variety of genes known to be involved in chondrogenesis. The genes that met both criteria are listed in Table 1 and included transcription factors (*Sox9*, *Sox6*, *Atf4*, and *Runx2*), extracellular signaling molecules (*Ihh*, *Bmp7,* and *Pthrp*), hormone and growth factor receptors (*Pth1r* and *Fgfr3*), and cartilage matrix proteins (*Col2* [*Col2a1*], *Col10* [*Col10a1*], and *Agg*). Because we observed a reduction in proliferation within the proliferative zone of transgenic animals, and because ERRγ has been shown to suppress proliferation [21], we also assessed the expression of cell cycle regulators cyclin D1 (*CcnD1),* and cyclin dependant kinase inhibitor 1a and 1b (*Cdkn1a and Cdkn1b).* RT-qPCR revealed a significant increase in *Pth1r* (Figure 5A), but no differences in the transcription factors tested (Figure 5B). We also observed an increase in cartilage matrix proteins *Col2* and *Agg* (Figure 5C) and the cell cycle regulator *Cdkn1b (p27)* (Figure 5D) in Line 2 (higher overexpresser). This suggests that overexpression of ERRγ decreases chondrocyte proliferation through regulation of *Cdkn1b*, impacts growth plate organization, matrix synthesis as evidenced by upregulation of *Col2* and *Agg*, and affects chondrocyte maturation through upregulation of *Pth1r.*

**Table 1:** Results of *in silico* search for putative ERRE sites in known regulators of growth plate chondrocytes (RZ = resting zone; PZ = proliferative zone; PHZ = pre-hypertrophic zone; HZ = hypertrophic zone).

Gene	Expression	Function	Putative ERRE	Position (bp)
*Atf4*	PZ & PHZ	Proliferation	ACAAGGACA	−7522
			TTAAGGTCA	−6669
			ACAAGGACA	−3214
			CAAAGGTCA	−606
*Bmp7*	PHZ	Proliferation &	TAAAGGTCA	−4858
		Differentiation	TCTAGGTCA	−4235
			TAAAGGTCA	+1692
			CAAAGTTCA	+3245
			CTAAGGTCA	+4796
*CcnD1*	RZ, PZ, PHZ, HZ	Proliferation	TCGAGGTCA	−4175
			TCTAGGTCA	+1117
*Fgfr3*	PZ, PHZ, HZ	Proliferation &	GAGAGGTCA	−6661
		Differentiation	TGAAGGACA	+4463
*Ihh*	PHZ	Proliferation &	TAAAGGTCA	−5988
		Differentiation	TCAAGGTCA	−2716
			TCAAGGACA	+1353
			CAAAGGTCA	+4046
*Pth1r*	PZ, PHZ	Proliferation &	CAAAGGTCA	−9433
		Differentiation	CCAAGGTCA	−2698
*Runx2*	PHZ, HZ	Differentiation	CAAAGTTCA	−6895
			CAAAGGTCA	−3156
			CAAAGTTCA	+4145
*Sox6*	RZ, PZ, PHZ	Proliferation &	TCAAGGACA	−6264
		Differentiation	ATGAGGTCA	−2802
			TCAAGGTCA	−978
*Sox9*	RZ, PZ, PHZ	Proliferation &	CCAAGGTCA	−8797
		Differentiation	CCAAGGTCA	−2966
*Pthrp*	RZ, PZ, PHZ	Proliferation &	TTCAGGTCA	−2728
		Differentiation		
*Col2*	RZ, PZ, PHZ	Cell-matrix	TAGAGGTCA	−8714
		communication	CAGAGGTCA	−7002
			GTAAGGTCA	−2325

Position is relative to the start site.

## Discussion

We report here that overexpression of ERRγ2 in chondrocytes results in decreased axial and appendicular skeleton size and disruption of growth plate height and organization. This phenotype was manifested already in newborn mice, indicating an effect on embryonic skeletal development that persisted postnatally, and included changes in chondrocyte proliferation, differentiation, and maturation, as well as matrix production, suggesting a role for ERRγ in chondrogenesis.

In the two independent Col2::ERRγ2FL mouse lines analyzed, ERRγ2 is overexpressed at moderate levels (3 to 5 fold higher levels than endogenous ERRγ expression in WT littermates) and the resultant skeletal phenotype and concomitant changes in gene expression observed are relatively subtle and ERRγ dose-dependent. It therefore seems likely that the phenotype seen in Col2::ERRγ2FL mice is a direct consequence of modulation of ERRγ transcriptional activity rather than from disruption of the transcriptional machinery. Nevertheless, it remains possible that the effects of ERRγ2 overexpression are indirect, e.g., resulting from an imbalance in the ratio of the ERRγ1/ERRγ2 protein isoforms and sequestration of required transcription cofactors (see also below). Thus, further studies in ERRγ knockout mice, in particular chondrocyte-specific ERRγ knockout mice to circumvent the perinatal lethality seen in global ERRγ knockout mice ([14] and Cardelli and Aubin, unpublished data), and in Col2::ERRγ1 overexpressing mouse lines, are of interest.

The most pronounced effect observed in the Tg growth plate was the reduction in height of the proliferative zone, with a 22% reduction in proliferative zone length and a 30% decrease in the percentage of proliferative Tg compared to WT cells. Despite the fact that *CcnD1* contains 2 putative ERREs in its regulatory region (Table 1), we did not observe any difference in *CcnD1* expression in WT versus Tg 14.5 dpc limbs. However, we observed increased expression of the cyclin-dependent kinase inhibitor *Cdkn1b* in Tg limbs, suggesting regulation of chondrocyte proliferation through regulation of this kinase inhibitor, a possibility consistent with data showing that ERRγ suppresses S-phase progression in an *in vitro* model of prostate cancer through transactivation of *Cdkn1a* and *Cdkn1b* [21]. A scan of the *Cdkn1b* regulatory region reveals 3 putative ERRE binding motifs at positions −5077, −2625, and −2618, suggesting potential direct regulation, however we cannot rule out a novel, indirect protein interaction.

In addition to the changes in cell cycle regulators and proliferation, we observed slightly reduced hypertrophic zone length in some bones, marked disorganization of the proliferative and hypertrophic zones, and acellular swaths that spanned a large portion of the Tg growth plate, which were not due to an increase in apoptosis. Moreover, *Col2, Agg,* and *Pth1r* expression were increased in 14.5 dpc Col2::ERRγ2FL mice. Taken together, the data suggest that ERRγ plays a role in coordinating chondrocyte proliferation-differentiation-matrix synthesis, but whether the changes are directly and causally related remain to be determined. Nevertheless, increased COL2, a cartilage extracellular matrix (ECM) protein that binds to integrins and activates signaling pathways essential to chondrocyte proliferation, may contribute to both acellular swaths of cartilage and decreased chondrocyte proliferation, via a negative feedback loop between abundant matrix production and proliferation. For example, it has been shown in chondrocytes from mouse ribcage that lack of β1 integrin results in reduced chondrocyte motility and COL2 adhesion, as well as reduced *CcnD1*, and increased *Cdkn1a* expression, resulting in decreased chondrocyte proliferation [22]. Further, it has been shown that ERRα is involved in osteoclast migration and adhesion, in part through regulation of β3 integrin [23]. It is necessary to determine whether integrin expression and/or chondrocyte adhesion and motility are affected in the ERRγ-overexpressing mice.

Whether *Col2* is regulated directly by ERRγ or indirectly through interaction with a factor upstream of *Col2* remains to be determined. It has been shown that overexpression of NKX3.2 *in vitro* increases *Col2* expression in a SOX9-independent manner, by directly binding to a 48 bp chondrocyte-specific enhancer in the *Col2* regulatory region [24]. Scanning the regulatory region using a core ‘AGGTCA’ sequence reveals 3 putative ERREs through which ERRγ could directly control *Col2* expression. Alternatively, ERRγ may regulate *Col2* expression through the recruitment of cofactors, such as PGC1-α and CBP/P300, as has been shown in SOX9-dependent regulation of *Col2* [25], [26]. Interestingly, it has been shown that PGC1-α is a cofactor of ERRγ [27], and it is possible that ERRγ and SOX9, together with PGC1-α, are part of a larger transcriptional complex to regulate *Col2* expression. Intriguingly, one of the ERREs in the *Col2* regulatory region is near the SOX9 binding site, supporting the hypothesis of a larger transcriptional regulatory complex.

Analyses of several genetically-engineered mouse models have revealed the importance of FGF [28], [29] and IHH [3], [30], [31] signaling in chondrocyte proliferation and differentiation. Recently, it has been reported that several transcription factors regulate the transcription of *Ihh*, including RUNX2 [32], ATF4 [33], and MSX2 [34]. The phenotype we observed in the ERRγ-overexpressing mice did not result in transcriptional changes in *Ihh* or *Fgfr3*, suggesting either a regulatory mechanism involving a transcriptional complex with one of the above mentioned transcription factors or regulation independent of IHH or FGFR3.

It will also be important to further analyze how ERRγ regulates chondrocyte maturation and hypertrophy, as no significant changes were detected in the hypertrophy markers assessed, such as *Runx2*, or its target gene *Col10*. While this may reflect simply the small content of hypertrophic cells in the 14.5 dpc samples utilized, it is worth noting that we also detected no differences in *Col10* expression in 17.5 dpc or P7 bone samples (data not shown). This suggests that ERRγ may have only a small or secondary role in chondrocyte hypertrophy. Alternatively, the modest difference we observe in the Tg hypertrophic zone height may be too small to quantify significant differences in hypertrophic marker gene expression. As mentioned, the presence of the acellular region in the Tg animals, made measurement of the growth plate zones difficult, and may have influenced the observation of significant differences where observed. On the other hand, the increase in *Pth1r* expression seen in 14.5 dpc Tg animals may contribute to a reduction in the hypertrophic zone through delay in the differentiation process. *Pth1r* knockout mice exhibit shortened limbs characterized by decreased proliferating chondrocytes. However, they also display premature hypertrophy at early embryonic stages [35]. By contrast, transgenic mice that constitutively express *Pth1r* display a severe delay in the endochondral process, including a reduced zone of hypertrophy [36]. Although we did not observe any differences in hypertrophic gene expression in our mouse model, additional studies on the growth plate of early embryonic mice are needed to elucidate the basis of hypertrophic zone anomalies.

In addition to the above factors, estrogen and expression of soluble and membrane-bound estrogen receptors (ERs and GPR30, respectively) have also been implicated in regulating growth plate chondrogenesis. Mice expressing *Col2* promoter-driven ERα have reduced proliferation and differentiation, and subsequent dwarfism [6], while mice with cartilage-specific inactivation of ERα exhibit prolonged longitudinal bone growth [5]. Taken together, the evidence suggests that not only ERα, but also ERRγ are negative regulators of chondrocyte proliferation and differentiation, which appear to be opposite to the function of ERRα [16]. Further, ERRα is able to form heterodimers with ERRγ [37] and ERα [38], suggesting that regulation of chondrocyte proliferation and differentiation may require a carefully controlled balance of the nuclear receptors. It remains to be elucidated if any interaction occurs between ERRγ and ERRα during chondrogenesis. It is also possible that the high ERRγ2 expression in our model may turn ERR transcriptional activation into repression. It was demonstrated that ERRα and ERRγ can independently activate an ERRE-driven promoter reporter, but when heterodimers of ERRα/γ were formed, the same reporter was suppressed [37].

In summary, overexpression of ERRγ2 in a cartilage-specific manner leads to dose-dependent abnormalities in the axial and appendicular skeletons due to alterations in *Cdkn1b* expression and chondrocyte proliferation as well as differentiation-maturation- matrix synthesis. Work is ongoing to characterize further the mechanism by which ERRγ exerts its actions in the developing growth plate.

## Materials and Methods

### Ethics Statement

All experimental procedures were performed in accordance with protocols approved by the Canadian Council on Animal Care and the University of Toronto Faculty of Medicine and Pharmacy Animal Care Committee.

### Construction of pCol2a1mERRγ2 and Generation of Transgenic Mice

We made a generic transgene vector containing the mouse *Col2a1* promoter (gift from B. deCrombrugghe) and flanking intron [39] fused to a splice acceptor site [40] with stop codons in all reading frames, an extensive multiple cloning site (MCS), a Polio internal ribosome entry site (gift from P.A. Greer), a nuclear localized β-galactosidase [41] and the protamine minigene pA [42]. During the construction of this vector, silent mutations were introduced to abolish restriction sites within the transgene so that the MCS could be expanded for future cloning of cDNAs. The transgene is flanked by NotI restriction sites. The longer 458 amino acid open reading frame for ERRγ2 (NM_001243792) was PCR amplified from mouse muscle cDNA with primers containing restriction enzymes to facilitate cloning. All vectors were verified by sequencing. Transgenic lines carrying the pCol2a1mERRγ2 construct were generated by pronuclear injection, as described previously [43]. Hemizygous founders were screened for transmission of the transgene by performing PCR on DNA isolated from tail clips taken from the F1 generation of progeny using primers in the IRES (TGC TCC TTT GAA ATC TTG TGC A) and LacZ (AAG TTG GGT AAC GCC AGG GT) portion (Figure 1). To determine sex of the P0 or P7 pups, we performed PCR targeting the sex-determining region Y (*Sry*) (Table 2).

**Table 2:** Primer sequences used in gene expression analysis.

Gene	Upstream Sequence	Downstream Sequence
*L32*	CACAATGTCAAGGAGCTGGAAGT	TCTACAATGGCTTTTCGGTTCT
*Bmp7*	GCACTCAGGCAGGGAGTCGG	ACCCAGTGGTTGCTGGTGGC
*Ihh*	TGCTGGCGCGCTTAGCAGTG	GCAGCGGCCGAATGCTCAGA
*Pthrp*	ATTCCTACACAAGTCCCAGAG	ACTTGCCCTTGTCATGCAGTA
*Pth1r*	GACGTGGGCCAACTACAGCG	GTGCAGTGCAGCCGCCTAAA
*Fgfr3*	TAGCGGCCGCCAGTCTCCAC	ACGCAGGCCGGGACTACCAT
*Sox9*	AATGCTATCTTCAAGGCGCTG	GGACCCTGAGATTGCCCAG
*Sox6*	ACAACCACAGACAGATTGAGCAGC	TGCCCCTGCCGAGTTTGGTG
*Atf4*	ATGGCGTATTAGAGGCAGCA	GATTTCGTGAAGAGCGCCAT
*Runx2*	TGTTCTCTGATCGCCTCAGTG	CCTGGGATCTGTAATCTGACTCT
*Col2*	ACTGGTGGAGCAGCAAGAGC	TCTGGACGTTAGCGGTGTTG
*Col10*	AACGGTACCAAACGCCCAC	CTTTGTTCTCCTCTTACTGGAATCCC
*Agg*	GCGTGAGCATCCCTCAACCATC	GGCAGTGGTCACAGGATGCATG
*CcD1*	CCTGTGCGCCCTCCGTATCT	TCATGGCCAGCGGGAAGACC
*Cdkn1a*	CAGACCAGCCTGACAGATTTCTA	GAGGGCTAAGGCCGAAGATG
*Cdkn1b*	GTTTCAGACGGTTCCCCGAA	TCTTAATTCGGAGCTGTTTACGTC
*ERRα*	TCGAGAGATAGTGGTCACCATCAG	CTTCCATCCACACACTCTGCAG
*ERRβ*	TGAGATCACCAAACGGAGGC	GAACTCGGTCAAGGCGCA
*ERRγ*	TGTGACTTGGCTGACCGAGA	TGGAGGAGGCTCATCTGGTCT
*ERα*	GGCTGCGCAAGTGTTACGAA	CATTTCGGCCTTCCAAGTCAT
*ERβ*	TTGGTGTGAAGCAAGATCACTAGAA	GACTAGTAACAGGGCTGGCACAA
*Sry*	GAGAGCATGGAGGGCCAT	CCACTCCTCTGTGACACT

### LacZ Stain for Detection of Transgene

Embryos of 14.5 dpc were dissected, and processed for LacZ detection as previously described [44].

### Gene Expression Analysis

Embryonic tissues and adult articular knee cartilage were homogenized using an Ultra Turrax T25 homogenizer, while postnatal tissues were manually ground with a mortar and pestle under liquid nitrogen. The RNA was extracted using TRIzol (Invitrogen), precipitated in isopropanol, and resuspended in 50–200 µL of DEPC dH_2_O. To remove potential contaminating DNA, RNA samples were subjected to DNase treatment, using an Ambion Turbo DNA-Free kit (Invitrogen), as per the manufacturer’s directions. Three µg of DNased RNA were reverse transcribed using Superscript II Reverse Transcriptase (Invitrogen), according to the manufacturer’s directions. All primers were designed with intron inclusion in corresponding genomic DNA, and are common to all potential transcript variants (Table 2).

### Western Blotting

Dissected limbs in PBS were homogenized (Ultra Turrax T25), followed by lysis in RIPA buffer (50 mM Tris HCl pH 8, 150 mM NaCl, 1% NP-40, 0.5% sodium deoxycholate and 0.1% sodium dodecyl sulphate) with added protease inhibitors. Protein samples were quantified using the Bio-Rad DC Protein Assay kit, following the manufacturer’s instructions. Thirty µg of each sample was run in a 10% SDS-PAGE gel, transferred to polyvinylidene fluoride (PVDF) membrane, followed by blocking in 5% milk-TBS-T for 30 minutes at room temperature. Immunodetection was carried out using a rabbit polyclonal anti-ERRγ antibody (H38x, Santa Cruz Biotechnology Inc.) diluted 1∶5000 in blocking buffer, or rabbit anti-β-ACTIN antibody diluted to 1∶2000 (Sigma). This was followed by a one hour incubation with a goat anti-rabbit IgG, conjugated to horse radish peroxidase (HRP; Santa Cruz Biotechnology), diluted 1∶5000–1∶8000 in blocking buffer. The HRP was visualized using the Amersham ECL Western Blotting Detection kit (GE Healthcare), as per the manufacturer’s instructions. The autoradiographic films from both ERRγ and β-ACTIN detection were scanned and the density and size of the bands were quantified using Image Lab software (Bio-Rad). The ERRγ band was normalized to the β-ACTIN band to assess proportionate protein levels. The 29 amino acid difference between the ERRγ1 and ERRγ2 protein isoforms is not clearly obvious in the gels that we run and we attribute the increased expression we see to the ERRγ2 isoform.

### Whole Mount Skeletal Staining

P0 and P7 animals were dissected, eviscerated, and fixed in 95% ethanol overnight or up to two weeks, and then processed for whole mount skeletal staining as previously described [45]. When samples were fully cleared, skeletons were dissected and photographed in a Petri dish containing 100% glycerol, using a Nikon Coolpix P5100 digital camera affixed to a dissecting scope. The images were then quantified in Image J by taking linear measurements of individual skeletal components.

### Histological and Immunofluorescence Analysis

The left limbs taken from each skeleton analyzed by whole mount staining were fixed in 4% paraformaldehyde for 24 hours, transferred to PBS for 2–3 days and then decalcified (10% EDTA, 0.1 M Tris pH 7.4) for 15 days. Once decalcified, limbs were serially dehydrated with one 20 minute wash in 30% ethanol, three 20 minute washes in 50% ethanol and a final wash and storage in 70% ethanol. Samples were paraffin-embedded, sectioned (5 µm), and stained (hematoxylin and eosin, and Safranin-O) at the Toronto Centre for Phenogenomics (TCP). The Safranin-O-stained slides were photographed under a Nikon Eclipse TS100 fluorescence microscope, at magnifications of 40X and 100X. In the 40X micrographs, measurements were taken from the absolute edge of Safranin-O staining before the articular surface, down to the last hypertrophic chondrocyte before the primary spongiosa. Three such measurements were taken at separate axes along the bone, with the average representing the total growth plate height. Using the 100X micrographs, measurements were taken from the first flattened chondrocyte, which appeared in a distinct column of three or more cells, down to the last hypertrophic chondrocyte, and then the subset of that distance that contained only hypertrophic chondrocytes was taken. This was repeated at five points along the width of each growth plate, and the averages were used to calculate the specific heights of the resting, proliferative and hypertrophic zones. For each animal, growth plates were assessed from the proximal humerus, distal femur and proximal tibia.

To immunodetect Ki67, a common proliferation marker [42], sections were deparaffinized and rehydrated in ethanol washes, followed by antigen retrieval in boiling citrate buffer (10 mM citric acid, 0.05% Tween-20, pH 6) for up to 20 minutes. The slides were blocked in normal goat serum (Invitrogen) for 30 minutes at room temperature, washed, incubated with rabbit polyclonal anti-Ki67 antibody (diluted 1∶25 in blocking buffer) for 1 hour at room temperature, washed, then incubated with goat anti-rabbit Alexa-594-conjugated secondary antibody (Invitrogen, diluted 1∶50 in blocking buffer) for 30 minutes at room temperature. The samples were counterstained with Hoechst nuclear stain.

Growth plate sections of the proximal humerus were viewed and imaged using a Bioquant Osteo imager with Photofluor II fluorescence excitation light, a triple Chroma filter, and Bioquant Osteo 2012. Ki67 positive and negative cells within the proliferative and resting zones were counted in Image J. By calculating the percentage of total cells (defined by the Hoechst stain) that stained positively for Ki67, the mitotic activity in the proliferative zone and resting zone was measured.

### TUNEL Assay

Growth plate sections of the proximal humerus were processed as described above, before using the FragEL DNA fragmentation detection kit (Calbiochem), as per the manufacturers instructions, and counterstained with methyl green. Sections were imaged at 16X magnification, using a Nikon Eclipse TS100 fluorescence microscope. Images were analyzed using ImageJ. Cells within the proliferative and hypertrophic zones were counted for TUNEL analysis and normalized to methyl green stained nuclei.

### Expression Analysis of Putative Target Genes

A list of putative target genes was constructed by *in silico* analysis of the regulatory regions of factors known to be involved in the chondrocyte differentiation process and that contained at least one ERR binding site (ERRE) in the region from −10 Kb to +5 Kb of their annotated transcription start site. The search used the ERRE consensus sequence TCAAGGTCA and 20 additional sequence variants that had been identified in the literature [46]. The search was performed using the freely available Transcriptional Regulatory Element Database (TRED) (http://rulai.cshl.edu/cgi-bin/TRED/tred.cgi?process=home), which retrieves genomic sequences from the current Ensembl build of the mouse genome. The results from this search are shown in Table 1, along with the primers used to look for differences in gene expression in Table 2. The primers were chosen to pick up all of the known transcript variants and include at least one intron in the corresponding genomic DNA.

The reactions were performed in triplicate on a 96-well plate in a BioRad MyIQ iCycler, for 50 cycles with an annealing temperature of 59°C. The amplification data was uploaded into the PCR miner program (http://www.ewindup.info/miner/version2/) to obtain the Ct and reaction efficiency values. The relative expression levels of the target gene were normalized to the *L32* internal control expression.

### Statistical Analysis

All data were analyzed using Graphpad Prism 4.0 software. When three data sets were analysed, ANOVA was used first to determine significance, followed by Student’s t-Test. All the graphs are plotted as the mean ± standard deviation and the p values listed are for the comparison to the WT values. Graphs were constructed using Microsoft Excel 2003 software.

### Mouse Gene Nomenclature

We followed the mouse nomenclature guide as stated on the Mouse Genome Informatics web page (http://www.informatics.jax.org/mgihome/nomen/short_gene.shtml). Thus, mouse genes are written with first letter capitalized followed by small letters, all italicized, while proteins are written all capitalized and without italics.
